# Enhanced Simultaneous Localization and Mapping Algorithm Based on Deep Learning for Highly Dynamic Environment

**DOI:** 10.3390/s25082539

**Published:** 2025-04-17

**Authors:** Yin Lu, Haibo Wang, Jin Sun, J. Andrew Zhang

**Affiliations:** 1 School of Internet of Things, Nanjing University of Posts and Telecommunications, Nanjing 210003, China; luyin@njupt.edu.cn (Y.L.); 1022072001@njupt.edu.cn (H.W.); sunjin@njupt.edu.cn (J.S.); 2 Global Big Data Technologies Centre (GBDTC), University of Technology Sydney (UTS), Sydney, NSW 2007, Australia

**Keywords:** deep learning, simultaneous localization and mapping, YOLOv10n, semantic segmentation

## Abstract

Visual simultaneous localization and mapping (SLAM) is a critical technology for autonomous navigation in dynamic environments. However, traditional SLAM algorithms often struggle to maintain accuracy in highly dynamic environments, where elements undergo significant, rapid, and unpredictable changes, leading to asymmetric information acquisition. Aiming to improve the accuracy of the SLAM algorithm in a dynamic environment, a dynamic SLAM algorithm based on deep learning is proposed. Firstly, YOLOv10n is used to improve the front end of the system, and semantic information is added to each frame of the image. Then, ORB-SLAM2 is used to extract feature points in each region of each frame and retrieve semantic information from YOLOv10n. Finally, through the map construction thread, the dynamic object feature points extracted by YOLOv10n are eliminated, and the construction of a static map is realized. The experimental results show that the accuracy of the proposed algorithm is improved by more than 96% compared with the state-of-the-art ORB-SLAM2 in a highly dynamic environment. Compared with other dynamic SLAM algorithms, the proposed algorithm has improved both accuracy and runtime.

## 1. Introduction

Simultaneous localization and mapping (SLAM) [1] is an enabling technology for many robot applications, such as collision-free navigation [2], environmental monitoring [3], warehousing and logistics [4], virtual reality [5], and agricultural automation [6]. The SLAM algorithm can estimate the map of an unknown environment and the robot’s posture on the map according to the data flow of the airborne sensor. The SLAM algorithm enables the robot to continuously locate itself in the same environment without accumulating drift, which is in stark contrast to the odometer method [7]. The odometer method integrates the estimated incremental motion into a local window, which makes the odometer still unable to correct the drift even if it passes through a certain position again.

Vision-based SLAM technology has received great attention in the past several years. The SLAM algorithm can be divided into sparse, semi-dense, and dense according to the pixels used. The common schemes are Semi-Direct Monocular Visual Odometry (SVO) [8], LiDAR SLAM and Detection (LSD) [9], and so on.

At present, visual SLAM technology assumes that the experiment is carried out in a static scene [10,11], which poses strict limitations on the application environment. When the SLAM algorithm is applied to a dynamic environment, dynamic objects are usually classified as abnormal data, so they are neither used for camera tracking nor included in the map. To handle this problem, outlier removal algorithms, such as the Random Sample Consensus algorithm (RANSAC) [12], can be applied.

Among many SLAM algorithms with RANSAC, the most widely used is ORB-SLAM2 [13]. On the basis of ORB-SLAM1, ORB-SLAM2 is extended by adding support for the binocular camera and RGB-D camera, which enables the algorithm to perform 3D reconstruction in more complex scenes. In addition, it also improves the performance of closed-loop detection and map optimization, so that the algorithm can achieve efficient positioning and mapping when different types of cameras are used.

In the research of visual SLAM algorithms, there are many challenges in detecting and processing dynamic objects, including: How to avoid tracking algorithms using the features of dynamic objects to match? How to prevent the mapping algorithm from including the feature points of dynamic objects in the constructed map?

Recent SLAM algorithms applied in dynamic scenes mainly include two categories: one is dynamic SLAM based on object detection and semantic segmentation, and the other is dynamic SLAM based on optical flow and scene flow motion segmentation. The first category is the current mainstream solution of dynamic SLAM, which separates dynamic objects and static objects by semantic segmentation of objects in dynamic scenes. For example, Bescos et al. [10] used the Mask R-CNN target detection algorithm based on a deep neural network to eliminate dynamic feature points and then used the remaining static feature points for tracking and reconstruction. The second category uses optical flow information and scene flow information to segment objects in dynamic scenes, so as to achieve more accurate positioning and mapping. Gao et al. [14] proposed RTD-SLAM based on YOLOv5-LITE, which uses semantic information and optical flow to extract dynamic feature points.

However, despite the advancements in SLAM technology, several critical challenges remain unresolved, particularly in highly dynamic environments. A highly dynamic environment refers to the large, rapid, and unpredictable changes in various elements in the environment, which leads to the asymmetry of information acquisition. Existing SLAM algorithms, such as DynaSLAM and RTD-SLAM, face the following limitations:

Poor Real-Time Performance: Algorithms like DynaSLAM rely on computationally expensive methods such as Mask R-CNN for dynamic object detection, which significantly reduces their real-time performance.

Low Positioning Accuracy: In highly dynamic environments, the optical flow method used in RTD-SLAM struggles to accurately distinguish and estimate the motion of each object, resulting in poor positioning accuracy.

Limited Robustness: The robustness of existing algorithms is compromised when dealing with complex and fast-moving dynamic objects, leading to frequent failures in map construction and localization.

These limitations severely restrict the practical application of SLAM technology in real-world scenarios, such as autonomous navigation in crowded urban environments or dynamic industrial settings. Therefore, there is an urgent need to develop a SLAM algorithm that can address these challenges while maintaining high accuracy, real-time performance, and robustness.

In summary, the visual SLAM technology in a highly dynamic environment faces problems such as poor real-time performance, low positioning accuracy, and poor robustness. To overcome these problems, we use YOLOv10 to enhance the ability of ORB-SLAM2 to eliminate outliers in this paper, so as to establish a more accurate static map. The main contributions of this paper are as follows:

(1) A SLAM algorithm based on deep learning for applications in a highly dynamic environment is designed. Based on ORB-SLAM2, a process of segmenting dynamic targets using lightweight YOLOv10n is added. The process runs concurrently with the local mapping thread of ORB-SLAM2;
(2) The client and server based on socket process communication are designed. In YOLOv10, the server is used to transmit the detection results to the client. In ORB-SLAM2, the client is used to receive the detection results from the server, and the detection results are passed into the feature point detection module to filter the dynamic feature points. After ensuring the consistency and compatibility of the two ends of the communication interface, the information transmission between different processes is realized;
(3) The experimental results show that the proposed algorithm greatly improves the accuracy of the ORB-SLAM2 algorithm, and the improvement rate is more than 96%. The processing speed is also improved compared with other dynamic SLAM algorithms. In this paper, the average processing time of each frame of the image is 0.0488 s, and the average processing speed is 20 frames per second.

## 2. Dynamic Visual SLAM Algorithm Framework

**Figure 1:**
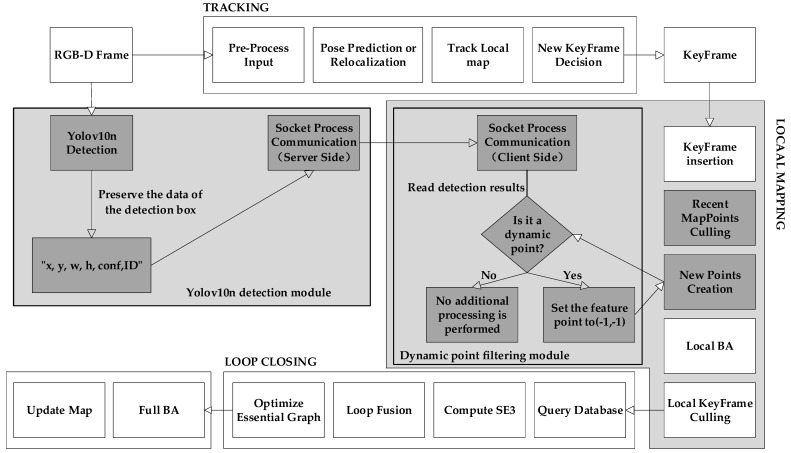
Algorithm flow chart.

The ORB-SLAM2 algorithm consists of three modules: tracking, local mapping, and loop closure detection [13]. The algorithm proposed in this paper is improved on the basis of ORB-SLAM2, and the overall flow chart of the algorithm is shown in Figure 1. The modules highlighted in gray are the ones added and optimized by the proposed algorithm.

**Figure 2:**
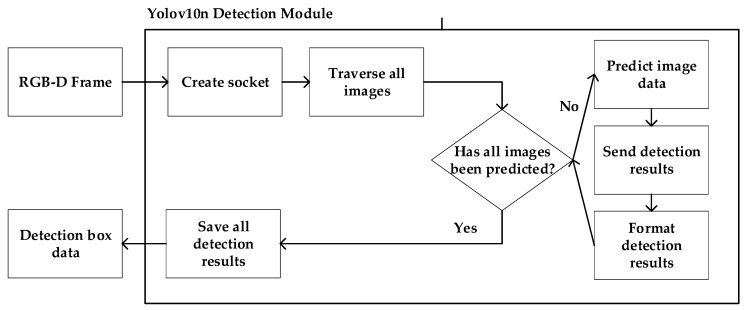
Yolov10n detection module.

In order to be able to work in a highly dynamic environment, the proposed algorithm adds a YOLOv10n detection module. The detection module and the local mapping module run concurrently. The Yolov10n detection module is shown in Figure 2. This module will traverse all the images in the dataset and output the data of the detection box. If the detection results show that the feature points are dynamic points, they are removed as outliers, and not retained.

**Figure 3:**
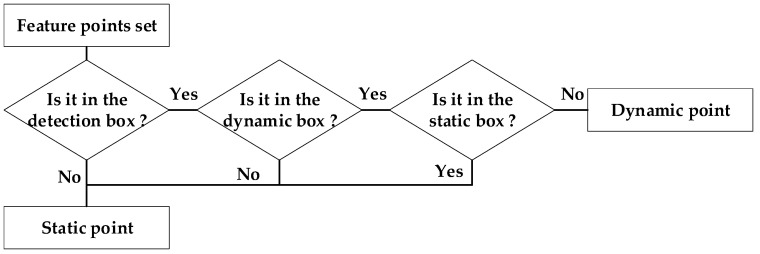
Dynamic point filtering module.

The specific methods are as follows: Firstly, the RGB-D image will be passed into YOLOv10 in timestamp order for detection. The results obtained by the detection only retain the detection box information. Then, the Socket communication connection is established to send the detection results to the local mapping process of ORB-SLAM2. After receiving the detection results, the local mapping thread tracks all the feature points of a frame of the image and uniformly detects whether they are in the frame of the dynamic target. If it is, the feature point will be marked as a bad point, and the outliers will be removed together in the subsequent steps. The dynamic point filtering module is shown in Figure 3. This method ensures the accuracy of the algorithm by maximally retaining static points and effectively eliminating dynamic points.

By introducing the YOLOv10n detection module, the proposed algorithm significantly reduces the dynamic object interference, thereby enhancing the accuracy and robustness of the algorithm in a highly dynamic environment.

### 2.1. Lightweight Target Detection

**Figure 4:**
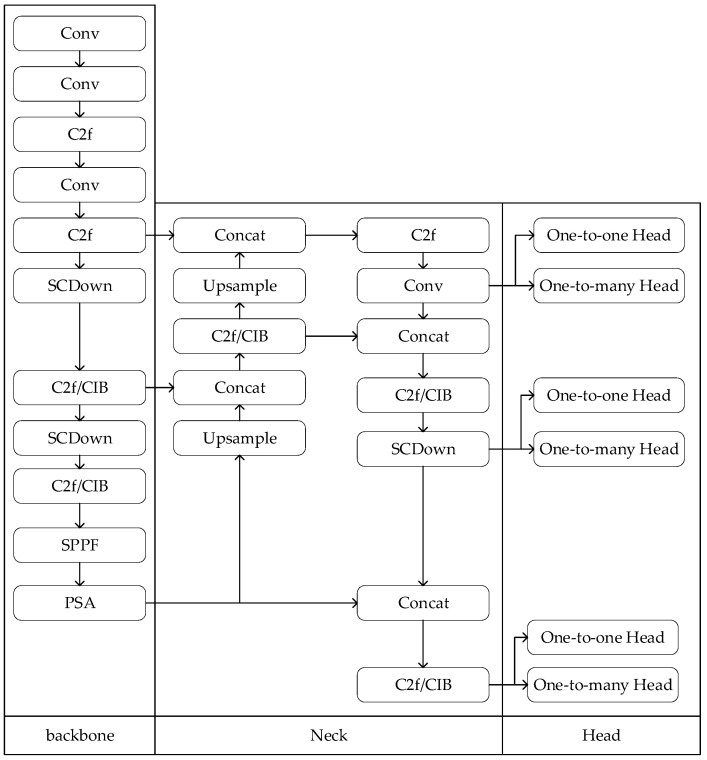
Network model diagram of YOLOv10n.

YOLOv10 is the latest target detection algorithm. In order to adapt to different tasks, YOLOv10 officially released six models: YOLOv10n, YOLOv10s, YOLOv10m, YOLOv10b, YOLOv10l, and YOLOv10x [15,16]. The model indicators are shown in Table 1. Because this paper has higher requirements for running time, according to the data in Table 1, YOLOv10n needs to calculate the least amount of parameters and the shortest time for target detection. Therefore, YOLOv10n is selected as the model of dynamic target detection. The network model of YOLOv10n is shown in Figure 4.

Compared with YOLOv8, the biggest change in YOLOv10 is the dual allocation strategy. During the training process, the model is used to jointly optimize the two heads, so that the backbone and neck enjoy the rich supervision signals provided by one-to-many tasks. In the reasoning process, the one-to-many strategy is discarded and the one-to-one strategy is used for prediction. This enables YOLO to perform end-to-end deployments without incurring any additional inference costs. The reason is that consistent matching metrics can be used, no matter whether it is a one-to-many strategy or a one-to-one strategy. During the execution of the program, both one-to-one and one-to-many strategies use the same indicator to quantitatively evaluate the level of consistency between the prediction and the instance. The matching measure used is as follows:

(1) $$m\left(\alpha ,\beta \right)=s\cdot p^{\alpha }\cdot IoU{\left(\hat{b},b\right)}^{\beta }$$

where p is the classification score, $\hat{b}$ and b are the bounding boxes of the prediction and the instance, respectively; s denotes a spatial prior that indicates whether the predicted anchor point is within the instance [16,17,18]. α and β are two hyperparameters used to balance the impact of semantic prediction tasks and location regression tasks on the results. The one-to-many and one-to-one metrics are denoted by the following:

(2) $$m_{o2m}=\left(\alpha_{o2m},\beta_{o2m}\right)$$

(3) $$m_{o2o}=\left(\alpha_{o2o},\beta_{o2o}\right)$$

These metrics will affect the label allocation and supervision information of the two strategies.

In the dual-label allocation, the one-to-many strategy provides richer monitoring signals than the one-to-one strategy. Intuitively, as long as one-to-one and one-to-many supervision can be coordinated, one-to-one strategies can be optimized in the direction of one-to-many. Therefore, a one-to-one head can provide higher-quality samples in the inference process, resulting in better performance. To this end, this paper first analyzes the gap between the supervision effects of the two strategies. Due to the randomness of the training process, the same value is initialized with two heads at the beginning, generating the same prediction. The one-to-one strategy and the one-to-many strategy generate the same p and IoU for each prediction instance pair. It can be observed that the mutually matched prediction targets are the same, and the mismatched prediction is ignored, so the regression targets of the two branches do not conflict. The difference in supervision effect lies in the different classification objectives. Given an instance, the maximum IoU is expressed as $u^{*}$, and the maximum one-to-many and one-to-one matching scores are expressed as $m_{o2m}^{*}$ and $m_{o2o}^{*}$, respectively. Assume that one-to-many branches produce a positive sample Ω, and the one-to-one branch selects the *i*-th prediction result by using the following metric:

(4) $$m_{o2m,i}{=m}_{o2o}^{*}$$

Then, for j∈Ω, it can be deduced that the classification objective is as follows:

(5) $$t_{o2m,j}=u^{*}\cdot \frac{m_{o2m,j}}{m_{o2m}^{*}}<u^{*}$$

For the task alignment loss, the classification goal can be derived as follows:

(6) $$t_{o2o,i}=u^{*}\cdot \frac{m_{o2o,i}}{m_{o2o}^{*}}=u^{*}$$

Therefore, the supervision gap between the two branches can be obtained by the first-order optimal transmission distance [19] of different classification targets:

(7) $$A=t_{o2o,i}-\prod \left(i\in \Omega \right)t_{o2m,i}+\sum_{k\in \Omega \{i\}}t_{o2m,k}$$

It can be concluded from Formula (7) that when i increases gradually in the range of i∈Ω, the spacing A decreases with the increase of $t_{o2m,i}$. When $t_{o2m,i}=u^{*}$, it reaches the minimum, that is, i is the best positive sample in Ω.

To achieve this goal, YOLOv10 uses a consistent matching metric, namely the following:

(8) $$\alpha_{o2o}=r\cdot \alpha_{o2m}$$

(9) $$\beta_{o2o}=r\cdot \beta_{o2m}$$

This means that $m_{o2o}=m_{o2o}^{r}$, indicating that the one-to-many best positive sample is also the one-to-one best positive sample. Therefore, the two strategies can be optimized in a consistent and coordinated manner.

The algorithm in this paper adds a module to extract the information of the detection box on the basis of YOLOv10. By retrieving the information of the specific address of YOLOv10, the number and confidence of the left boundary, upper boundary, right boundary, lower boundary, and object type in the coco dataset are obtained, respectively, and they are stored in the string result_to_orb. Then, through the function, the result_to_orb is encoded into the data type that the socket can transmit. Finally, the encoded target box information is transmitted to ORB-SLAM2 through the socket. The algorithm pseudo-code is shown in Algorithm 1.
**Algorithm 1** YOLOv10n Dynamic Detection AlgorithmInput: dataset_path: The path to the TUM dataset. model_path: The path to the YOLOv10n model. client_address: The address of the client. dynamic_object_id: ID of dynamic object. Output: detection_results: Detection box information Procedure:Load model from model_path.Load the dataset from dataset_path.Connection to client_address.For frame in the dataset_path: Predict(model_path, frame, 640, confidence). result_send = “x”. For box in frame.data:  result_for_k += (x, y, w, h, conf, ID).  If ID = dynamic_object_id:   result_to_orb += result_for_k. If result_to_orb! = NULL.  Send(result_to_orb.encode()). detection_results += result_to_orb.Return detection_results.

Compared with the Mask R-CNN target detection algorithm used by DynaSLAM, YOLOv10n is more efficient. Mask R-CNN has a computational complexity of $O(HW+Nm^{2})$, while YOLOv10n has O(HW). Where H*W is the image size, N is the number of RoIs, and m is the mask size. YOLOv10n does not require RPN or mask branches. Its total FLOPs are only 6.7 G, compared to 220 G + FLOPs for Mask R-CNN (ResNet-101). It is 30–35 times more efficient. It runs faster and is better for real-time applications. The YOLOv10 achieves high accuracy consistently. It also significantly reduces the algorithm’s time consumption.

YOLO-SLAM also uses YOLO for dynamic segmentation but adopts YOLOv3 with Darknet19 as the backbone. YOLOv3 has 25 G FLOPs and Ap^val^ of 34.9%. In contrast, the proposed algorithm achieves Ap^val^ of 38.5%. It improves both accuracy and speed.

RTD-SLAM makes it difficult to accurately distinguish and estimate the motion of each object in a highly dynamic environment, and YOLOv10 avoids this problem in principle. YOLOv10 is a semantic segmentation algorithm. The work is to classify the objects in the image without processing the motion information in the image. Therefore, no matter how complex the motion model in the image is, it will not increase the computational complexity of YOLOv10, and will not affect the real-time performance of the algorithm.

**Figure 5:**
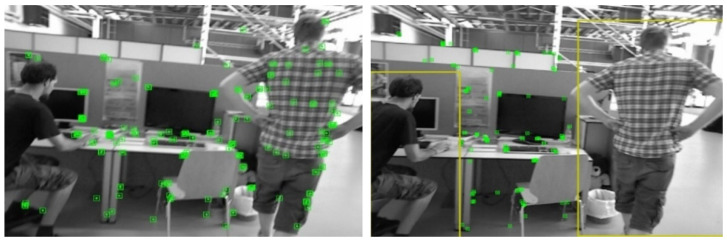
Comparison of the results before and after adding the YOLOv10 dynamic detection module.

The comparative SLAM results achieved by the algorithm are shown in Figure 5, for the cases before and after adding the YOLOv10 dynamic detection module. Before adding the module, relying only on ORB-SLAM2, the screening of dynamic points is almost ineffective. After adding the module, almost all dynamic points are identified, and the accuracy and robustness of the algorithm are greatly improved.

### 2.2. RANSAC + EPnP Algorithm

RANSAC is an iterative algorithm that can estimate the parameters of the mathematical model from a set of datasets containing outliers. The core of the algorithm is to assume that the dataset contains two types of data: internal points and external points, which can also be called normal and abnormal values. RANSAC uses a model with a specific parameter value to explain these data points, where the outliers do not conform to the assumed model in any case. In this way, RANSAC can separate normal and abnormal values during the estimation of the best model parameters.

**Figure 6:**
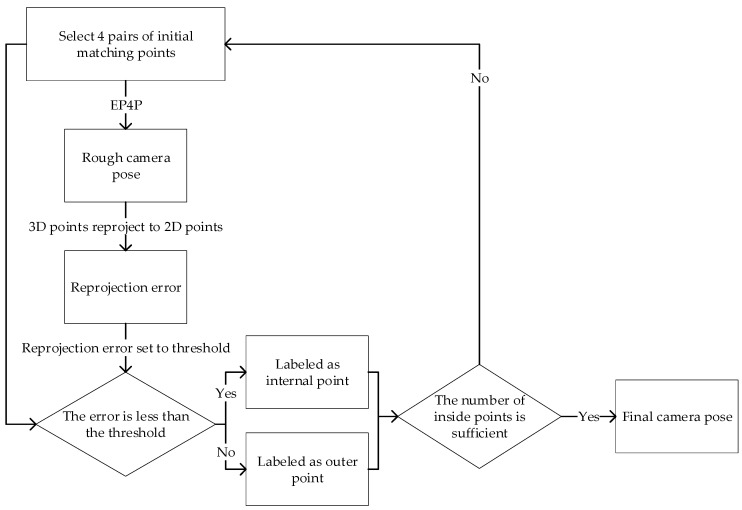
RANSAC + EPnP algorithm flow chart.

The main work of RANSAC is to randomly select a small number of sample points for model estimation. In order to improve the efficiency of the algorithm without affecting the accuracy of the RANSAC, this paper introduces the EPnP [20] algorithm. After many experiments, we set *n* = 4, that is, using the EP4P algorithm to optimize the RANSAC. The RANSAC + EP4P algorithm randomly selects 4 3D-2D matching point pairs from all feature points in each iteration. The purpose of selecting random point pairs is to ensure that even if there are mismatched point pairs, the real model can be found through multiple iterations. The algorithm flow chart is shown in Figure 6.

Firstly, four pairs of matching points are selected from the given 3D point set $X_{i}={\left[X_{i},Y_{i},Z_{i}\right]}^{T}$ and 2D point set $x_{i}={\left[u_{i},v_{i}\right]}^{T}$. Using the selected four-point pairs, the coarse pose of the camera is estimated by the RANSAC + EP4P algorithm. The purpose of this step is to quickly calculate a possible camera pose through a small amount of data, that is, to calculate a set of rotation matrix $R_{i}$ and translation vector $t_{i}$.

In order to project these four 3D points onto a 2D plane, it is necessary to know the camera’s internal parameter matrix *K* and the camera’s attitude relative to the world coordinate system $R_{i}$ and $t_{i}$. The projection relationship can be expressed as follows:

(10) $$s_{i}\left[\begin{array}{c} u_{i}\\ v_{i}\\ 1 \end{array}\right]=K\left(R_{i}X_{i}+t_{i}\right)$$

For each 3D-2D point pair, two independent scalar equations can be obtained. For 4 point pairs, a total of 8 equations about $u_{i}$, $v_{i}$ and $s_{i}$ are obtained. However, since $s_{i}$ is unknown and exists independently for each point, we use

(11) $$s_{i}=r_{31}X_{i}+r_{32}Y_{i}+r_{33}Z_{i}+t_{3}$$

to replace it. In this way, four independent equations can be obtained from four-point pairs, which can be expressed as a linear equation set to solve the unknown variables $R_{i}$ and $t_{i}$ in the camera pose. The EP4P algorithm solves these equations by the least square method to calculate $R_{i}$ and $t_{i}$. Through further iterative optimization, a rough camera pose prediction can be obtained.

Based on the predicted camera pose, the 3D points in all 3D-2D point pairs are re-projected into 2D points and the re-projection error of all points is calculated. Then, the inner points are determined based on the re-projection error. The inner point regards the reprojection error as a threshold, and the reprojection error is expressed as follows:

(12) $${ERROR}_{i}={\left\|x_{i}-\frac{1}{s_{i}}K\left(R_{i}X_{i}+t_{i}\right)\right\|}_{2}$$

The points less than the threshold are set as inner points, and the rest are regarded as outer points.

Next, the number of interior points needs to be counted. When the number of interior points meets the requirements, the camera pose will be solved again. After extensive computer experiments and parameter adjustments, the judgment condition is set as follows: when the proportion of matching interior point pairs reaches 70% and the total number is at least 15 pairs. This ensures that the algorithm remains reliable by preventing inaccuracies caused by a low proportion of matching point pairs while also avoiding the excessive elimination of valuable information due to an overly strict threshold. According to the latest solved $R_{i}$ and $t_{i}$, a set of interior point sets is calculated, and then the second RANSAC + EP4P solution is performed in this set of interior point sets to obtain an accurate camera pose.

### 2.3. Socket Process Communication

The algorithm proposed in this paper needs to transfer data between YOLOv10 and ORB-SLAM2, and they are developed by Python 3.9 and C++ 11, respectively. Therefore, this paper uses Socket process communication which can realize process communication between different programming languages.

**Figure 7:**
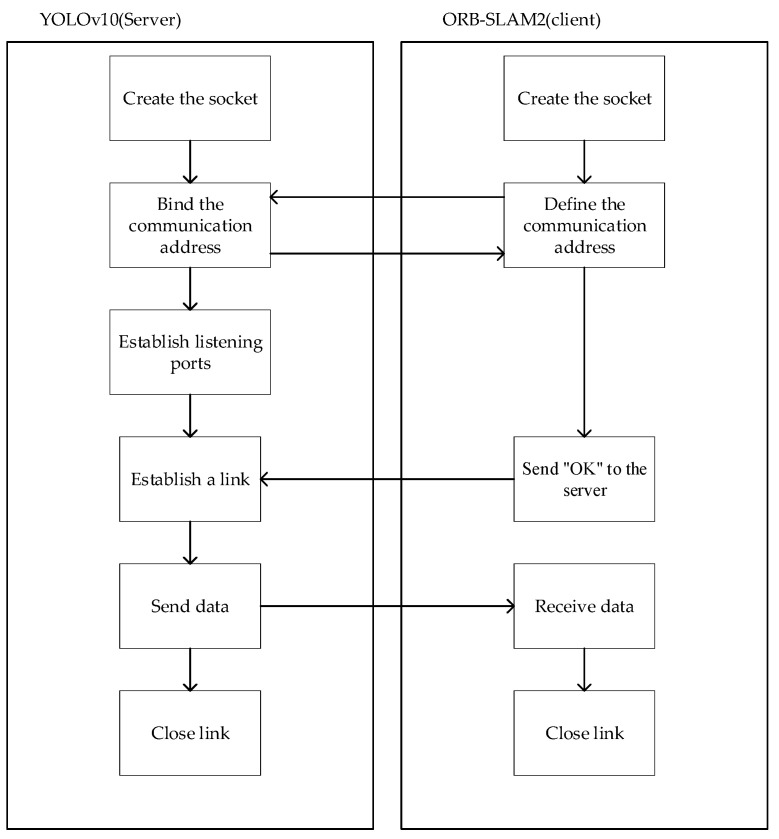
Socket communication module flow chart.

The principle of the socket module of the algorithm in this paper is shown in Figure 7. First of all, the server and the client will create a socket, and then the client defines the communication address, and the server binds. Then, the server will establish a listening port to detect the start signal sent by the server. The starting signal of the algorithm is ‘OK’. After the server receives the signal, it means that the connection has been successfully established, and then the result of dynamic target detection will be continuously sent to the client. After all the detection results are transmitted to the client, the connection will be closed and the operation of the Socket communication module will be ended.

YOLOv10 and ORB-SLAM2 are connected by Socket. In order to complete the transmission of information, this paper defines two local variables result_for_k and result_to_orb on the server side. Result_for_k stores the information of a detection box in the image, and the storage format is ‘left:{left} top:{top} right:{right} bottom:{bottom} class:{class_ids[class_id]} {confidence}*’, for example: left:100 top:150 right:200 bottom:250 class:person 0.87 *, where ‘*’ is the end tag of each detection box information. The result_to_orb stores all the detection box information of each frame image. For example: ‘x left:100 top:150 right:200 bottom:250 class:person 0.87 * left: 300 top:350 right:400 bottom:450 class:person 0.92 *’. The specific method is to splice the result_for_k stored in the dynamic target detection box behind result_to_orb. When all the information of a frame of image is stored in result_to_orb, the information will be transmitted to ORB-SLAM2 according to Figure 7.

In this paper, we define a local variable result_orb of vector < std::pair < vector < double>,int> > type in ORB-SLAM2, which is used to receive the detection box information. Result_orb only reads the information of one detection box from result_to_orb at a time. This paper also defines the global variable result_num, which is used to record the end address of each reading information, so that each time ‘*’ stops reading and records the address at this time. When preparing to read the next detection box, it will start from the next bit of result_num until all the detection boxes in a frame of the image are processed. The client resets result_num every time it receives a message from the server.

## 3. Experiment and Result Analysis

Since the processing speed of YOLOv10n for each picture significantly exceeds that of ORB-SLAM2, the overall time consumption of the program is greatly affected by the running time of ORB-SLAM2 after the dynamic detection module and Socket communication module are added to the whole algorithm. For the entire algorithm, the reason for the increase in running time is mainly due to the removal of dynamic target feature points and the transmission of information. The YOLOv10n dynamic detection module has little effect on the overall time consumption of the algorithm.

This paper evaluates the system in the public dataset TUM and compares it with other advanced dynamic SLAM algorithms in a highly dynamic environment. In addition, this paper also compares the proposed algorithm with the original ORB-SLAM2 to quantify the improvement of the accuracy, stability, and running speed of the proposed algorithm in highly dynamic scenarios.

**Figure 8:**
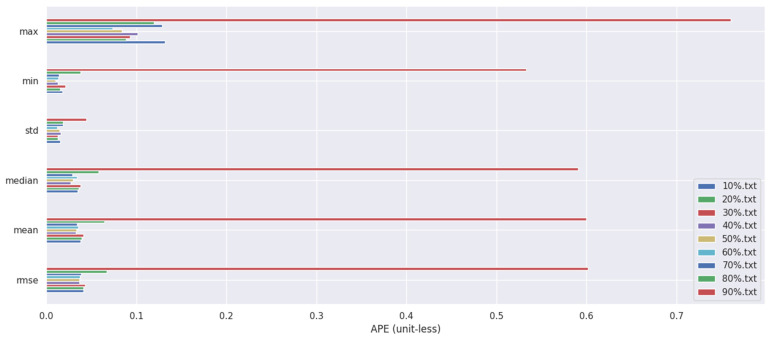
Algorithm performance under different confidence thresholds.

When running the proposed algorithm, we set a minimum confidence threshold for the algorithm. If the significance level is α, the confidence level is $\left(1-\alpha \right)$. A detection bounding box is considered valid only if its confidence exceeds $\left(1-\alpha \right)$. To determine the optimal value for the minimum confidence threshold, we conducted nine sets of experiments, increasing the minimum confidence from 10% to 90% in increments of 10%. The experimental results are shown in Figure 8.

It can be observed that as the confidence level gradually increases, the error initially decreases and then rises, reaching its lowest point at a confidence level of 50%, which corresponds to the highest accuracy. This is primarily because when the confidence level is too low, an excessive number of non-dynamic objects are incorrectly identified as dynamic, resulting in too few feature points available for trajectory prediction and thus reducing localization accuracy. On the other hand, when the confidence level is too high, some dynamic objects such as partially visible humans may fail to be detected, causing their feature points to be mistakenly treated as static points, which in turn interferes with the algorithm’s precision.

**Figure 9:**
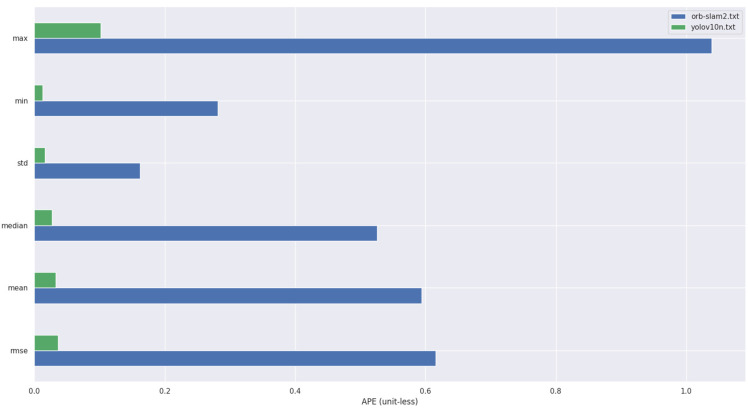
Comparison of errors between ORB-SLAM2 and the proposed algorithm on fr3/walking_halfsphere.

To intuitively demonstrate the extent of improvement achieved by our proposed algorithm compared to ORB-SLAM2, we collected data from running both our algorithm and ORB-SLAM2 on the fr3/walking_halfsphere dataset and plotted the results in a bar chart, as shown in Figure 9.

**Figure 10:**
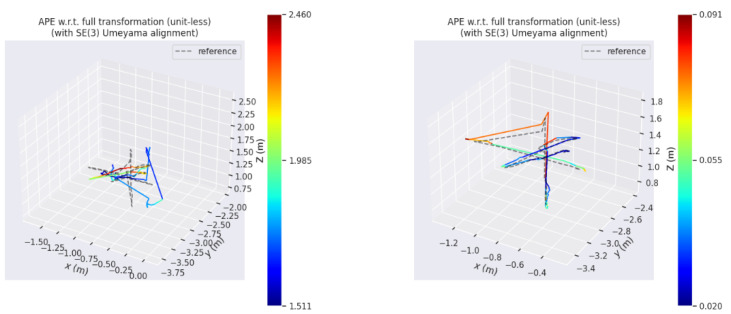
The absolute trajectory error of ORB-SLAM2 and the proposed algorithm on fr3/walking_xyz.

**Figure 11:**
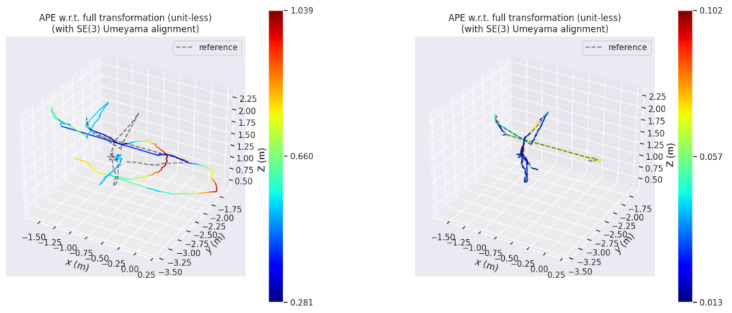
The absolute trajectory error of ORB-SLAM2 and the proposed algorithm on fr3/walking_halfsphere.

In this paper, the absolute trajectory root mean square error (RMSE) proposed by Sturm et al. [21] is used as the error measure of the experiment. In Figure 9 and Figure 10, the picture on the left side is the absolute trajectory error map of ORB-SLAM2, and the right side is the absolute trajectory error map of ORB-SLAM2 algorithm with YOLOv10n dynamic detection. The gray dotted line in the map is the real trajectory map, and the color real line is the predicted trajectory map. The two lines are closely fitted and the color is biased towards blue, indicating that the higher the accuracy of the algorithm. In addition, the errors represented by the same color in Figure 10 and Figure 11 are different. It can be observed that the error of ORB-SLAM2 is far more than the algorithm in this paper.

The datasets used for test are fr3/walking_xyz, fr3/walking_rpy, fr3/walking_static and fr3/walking_halfsphere. The four datasets correspond to different camera motion modes when collecting datasets, which are moving along the xyz axis, moving around the Euler angle rpy, stationary, and performing the semi-circular motion. These four datasets contain almost most of the scenes that visual SLAM can encounter in a dynamic environment.

In order to quantify the accuracy and stability improvement rate of the proposed algorithm, the improvement efficiency η is calculated as follows:

(13) $$\eta =\frac{y-x}{y}$$

where y represents the accuracy of the unoptimized ORB-SLAM2 algorithm, and x represents the accuracy of the proposed algorithm. After integrating the YOLOv10 dynamic target detection algorithm, the absolute trajectory error shows a significant improvement compared to ORB-SLAM2. We use RMSE to quantify the accuracy of the algorithm and the standard deviation (S.D.) to measure its stability. After calculation, it achieves an accuracy improvement of over 96% on all four datasets. Compared with ORB-SLAM2, the stability of the algorithm is improved by more than 78.5% on average. Detailed data are shown in Table 2. The values shown in bold in the table are the optimal values.

In addition, the proposed algorithm is compared with advanced algorithms such as DynaSLAM, RDS-SLAM, DS-SLAM, YOLO-SLAM, and RTD-SLAM. The results are shown in Table 3. The thickened part in the table is the optimal value.

It can be seen from Table 3 that the proposed algorithm performs best in fr3/walking_xyz, fr3/walking_static, and fr3/walking_halfsphere, and its accuracy is second only to DynaSLAM in fr3/walking_rpy. The stability of the proposed algorithm is comparable to that of other state-of-the-art methods.

The hardware environment used in this experiment is: CPU model is 12th Gen Intel (R) Core (TM) i7-12700H, with 14-core processing power, GPU model is Intel (R) Iris (R) Xe Graphics, with 8 GB video memory, system environment is Ubuntu20.04, programming languages are Python and C++. Table 4 shows the time consumed by various SLAM algorithms to process each frame of image in the hardware environment of this paper.

It can be seen from Table 4 that the time overhead of DynaSLAM is too large to realize real-time mapping and positioning, while the processing time of DS-SLAM, RDS-SLAM, and YOLO-SLAM is slightly reduced, but it is still much worse than the algorithm proposed in this paper. The algorithm in this paper takes an average of 0.0488 s to process each frame of image and can process 20 frames of image per second. Compared with others, the proposed algorithm shows better real-time performance.

## 4. Conclusions

In this paper, a visual SLAM algorithm for highly dynamic environments is proposed. The algorithm takes ORB-SLAM2 as the core and uses YOLOv10 to optimize the feature point detection function so that it can filter out static targets in complex and diverse dynamic targets and establish static sparse maps. In order to verify the performance of the algorithm, the open-source dataset TUM is used to comprehensively evaluate its accuracy and running time. The experimental results show that compared with other dynamic SLAM algorithms, the proposed algorithm achieves excellent performance stability in highly dynamic environments. It not only achieves high accuracy in terms of absolute trajectory estimation error, but also greatly reduces running time, fully proving the efficiency and robustness of the algorithm. In the future, visual–inertial odometry will be added to deal with the environment where visual SLAM algorithms face challenges, such as poor lighting conditions, lack of texture, rapid movement, or lens jitter, so as to further improve the accuracy and robustness of the SLAM algorithms.

## Figures and Tables

**Table 1:** Comparison of different YOLOv10 models.

Model	Test Size	#Params	FLOPs	Ap^val^	Latency
YOLOv10n	640	2.3 M	6.7 G	38.5%	1.84 ms
YOLOv10s	640	7.2 M	21.6 G	46.3%	2.49 ms
YOLOv10m	640	15.4 M	59.1 G	51.1%	4.74 ms
YOLOv10b	640	19.1 M	92.0 G	52.5%	5.74 ms
YOLOv10l	640	24.4 M	120.3 G	53.2%	7.28 ms
YOLOv10x	640	29.5 M	160.4 G	54.4%	10.70 ms

**Table 2:** Comparison of RMSE and S.D. between YOLOv10n-SLAM and ORB-SLAM2. (Unit: m).

	xyz		Static		rpy		Half	
	RMSE	S.D.	RMSE	S.D.	RMSE	S.D.	RMSE	S.D.
ORB-SLAM2	1.886891	0.283696	2.760182	0.004479	2.480451	0.230039	0.615633	0.162303
**Ours**	**0.0** **12961**	0.015896	**0.** **005218**	0.002650	**0.** **008292**	0.026500	**0.0** **20821**	0.015988

**Table 3:** YOLOv10n-SLAM is compared with other SLAMs. (Unit: m).

	DynaSLAM		RDS-SLAM		DS-SLAM		YOLO-SLAM		RTD-SLAM		Ours	
Sequence	RMSE	S.D.	RMSE	S.D.	RMSE	S.D.	RMSE	S.D.	RMSE	S.D.	RMSE	S.D.
w_xyz	0.015	0.009	0.057	0.023	0.025	0.016	0.015	0.007	0.020	0.009	**0.012**	0.015
W_rpy	**0.035**	0.019	0.160	0087	0.444	0.235	0.216	0.100	0.167	0.030	0.082	0.026
W_static	0.006	0.003	0.021	0.012	0.007	0.004	0.007	0.004	0.121	0.002	**0.005**	0.002
W_half_sphere	0.025	0.016	0.087	0.045	0.031	0.016	0.028	0.014	0.028	0.025	**0.020**	0.015

**Table 4:** Comparison of processing time. (Unit: s).

SLAM Algorithm	Processing Time per Frame
ORB-SLAM2	0.0239
DynaSLAM	>13
DS-SLAM	0.0793
RDS-SLAM	0.2137
YOLO-SLAM	0.7220
RTD-SLAM	0.0578
Ours	0.0488

## Data Availability

Data are contained within the article.
